# Chronic and postprandial effect of blueberries on cognitive function, alertness, and mood in participants with metabolic syndrome – results from a six-month, double-blind, randomized controlled trial

**DOI:** 10.1016/j.ajcnut.2023.12.006

**Published:** 2024-02-06

**Authors:** Peter J Curtis, Vera van der Velpen, Lindsey Berends, Amy Jennings, Laura Haag, Anne-Marie Minihane, Preeti Chandra, Colin D Kay, Eric B Rimm, Aedín Cassidy

**Affiliations:** 1 Nutrition and Preventive Medicine Group, Faculty of Medicine and Health Sciences, Norwich Medical School, University of East Anglia, United Kingdom; 2 Institute for Global Food Security, Nutrition and Preventive Medicine, School of Biological Sciences, Queen’s University Belfast, Northern Ireland; 3 Food Bioprocessing and Nutrition Sciences, Plants for Human Health Institute, North Carolina State University, North Carolina Research Campus, Kannapolis, NC, United States; 4 Department of Pediatrics, College of Medicine, University of Arkansas for Medical Sciences, Little Rock, AR, United States; 5 Departments of Epidemiology and Nutrition, Harvard T.H. Chan School of Public Health, and Channing Division of Network Medicine, Brigham and Women’s Hospital and Harvard Medical School, Boston, MA, United States

**Keywords:** cognitive function, metabolic syndrome, blueberry anthocyanins, anthocyanin-derived phenolic acid metabolites

## Abstract

**Background:**

Anthocyanin and blueberry intakes positively associated with cognitive function in population-based studies and cognitive benefits in randomized controlled trials of adults with self-perceived or clinical cognitive dysfunction. To date, adults with metabolic syndrome (MetS) but without cognitive dysfunction are understudied.

**Objectives:**

Cognitive function, mood, alertness, and sleep quality were assessed as secondary end points in MetS participants, postprandially (>24 h) and following 6-mo blueberry intake.

**Methods:**

A double-blind, randomized controlled trial was conducted, assessing the primary effect of consuming freeze-dried blueberry powder, compared against an isocaloric placebo, on cardiometabolic health >6 mo and a 24 h postprandial period (at baseline). In this secondary analysis of the main study, data from those completing mood, alertness, cognition, and sleep assessments are presented (i.e., *n* = 115 in the 6 mo study, *n* = 33 in the postprandial study), using the following: *1*) Bond-Lader self-rated scores, *2*) electronic cognitive battery (i.e., testing attention, working memory, episodic memory, speed of memory retrieval, executive function, and picture recognition), and *3*) the Leeds Sleep Evaluation Questionnaire. Urinary and serum anthocyanin metabolites were quantified, and apolipoprotein E genotype status was determined.

**Results:**

Postprandial self-rated calmness significantly improved after 1 cup of blueberries (*P* = 0.01; q = 0.04; with an 11.6% improvement compared with baseline between 0 and 24 h for the 1 cup group), but all other mood, sleep, and cognitive function parameters were unaffected after postprandial and 6-mo blueberries. Across the ½ and 1 cup groups, microbial metabolites of anthocyanins and chlorogenic acid (i.e., hydroxycinnamic acids, benzoic acids, phenylalanine derivatives, and hippuric acids) and catechin were associated with favorable chronic and postprandial memory, attention, executive function, and calmness.

**Conclusions:**

Although self-rated calmness improved postprandially, and significant cognition-metabolite associations were identified, our data did not support strong cognitive, mood, alertness, or sleep quality improvements in MetS participants after blueberry intervention.

This trial was registered at clinicaltrials.gov as NCT02035592.

## Introduction

Metabolic syndrome (MetS) affects around 1 in 3 adults [1,2] and predisposes to significant cardiovascular disease risk [[3], [4], [5], [6]], including accelerated incident cardiovascular disease in those aged <55 y [7]. MetS is also highly predictive of diabetes development, which has, in turn, been recognized as a mid-life modifiable risk factor for Alzheimer’s disease [8]. Likewise, an association between MetS and cognitive dysfunction has been frequently reported [9], especially in those with comorbid obesity [10] and within domains related to verbal fluency, processing speed, executive function, and verbal memory [11]. In brain imaging studies, MetS is associated with lower white and grey matter volumes [11,12] and disruption to cerebral homeostatic processes, including apoptosis, autophagy, and neurogenesis, in a manner consistent with neurodegeneration [13]. In one population-based study, MetS clinical criteria were inversely associated with grey matter volumes in the posterior cerebellum [14], a region shown to be functional during working memory, visual-spatial, and executive functioning tasks, and key to mood behavior, and frontal cognition [15,16]. These data illustrate a likely shared pathophysiology between vascular and cognitive health and identify the potential that dietary strategies that alleviate cardiovascular disease risk in MetS may also confer cognitive benefits.

In our previous publications from the **C**ardiovascular, **I**nsulin **R**esistance, **C**ognition and **L**ung function in m**E**tabolic **S**yndrome study (CIRCLES) trial (from which these current data are derived), we showed that freeze-dried blueberries (1 cup/d) improved 24 h postprandial and 6 mo chronic cardiometabolic health in participants with MetS [17,18]; supporting the benefits recognized in non-MetS populations [19]. Thus, increasing anthocyanin-rich blueberry intake is considered an exemplary dietary strategy to establish whether cognitive and cardiometabolic function benefits are aligned in MetS. In previous prospective cohort studies, habitual consumption of anthocyanin, the main flavonoid subclass in blueberries, has been associated with reduced subjective cognitive decline [[20], [21], [22]], and our UK Twins analyses confirmed that higher anthocyanin intakes were associated with greater executive function and simple reaction times, and larger left hippocampal volumes [23]. Similarly, for berry intake, the Nurses’ Health Study previously estimated that a higher habitual berry intake delayed cognitive aging by ≤2.5 y [20].

Across numerous randomized controlled trials in those with cognitive complaints [e.g., mild cognitive impairment (MCI), perceived/subjective cognitive decline], blueberry intake (12 wk to 6 mo) has improved speed of processing [24], executive function abilities [25], paired associate learning and word list recall [26] and cognitive symptoms and memory encoding during everyday activities [25,27]. Blueberry intake also increased blood oxygenation level-dependent activation in MCI (in the left precentral gyrus, middle frontal gyrus, and inferior parietal lobe) during working memory load tasks, indicating enhanced neuronal activation [28]. However, equivocal data have been observed in healthy populations without cognitive dysfunction. Although verbal learning appears enhanced following short-chronic blueberry intake in healthy populations (i.e., 3mo [[29], [30], [31]]), these effects have not been sustained at 6 mo [31], and domains, including reaction times, episodic memory, working memory, spatial memory, and executive function have been unaffected by blueberry intake [[30], [31], [32], [33]]. To date, only studies in healthy adults (i.e., without elevated cardiometabolic risk profiles) have assessed postprandial cognitive responses to blueberries, with cognitive benefits only attained during demanding and sustained tasks across this limited dataset [34,35]. Interestingly, blueberries have previously improved positive affect measures on the self-reported Positive and Negative Affect Schedule, suggesting mood improvement[36]; yet, this requires confirmation as other studies have not supported this effect [37]. To date, however, despite MetS being highly prevalent [1,2], cognitive function and mood data following postprandial or chronic blueberry intake in this large at-risk population subgroup are completely lacking.

In this analysis of the secondary end points of our CIRCLES trial, we, therefore, investigated the effects of 6-mo blueberry intake (at 2 dietarily achievable levels) on cognitive function, mood, and alertness in adults with MetS and also performed a 24 h postprandial assessment of the same endpoints in response to a single dose of the intervention material consumed as part of an energy-dense meal challenge.

## Methods

### Study design and participant population

A parallel, double-blind, placebo-controlled study was performed in adults aged 50–75y, with overweight and obesity [BMI (in kg/m^2^) ≥25], and MetS (≥3 MetS components, i.e., impaired fasting glucose, hypertension, central adiposity, hypertriglyceridemia, and low concentrations of HDL cholesterol [38]), but without self-reported cognitive dysfunction (assessed at health screening). A full description of the inclusion and exclusion criteria has been previously reported [18], with past or present smoking history, diabetes, vascular disease, cancer, neurologic, digestive, hepatic, renal disorders, or the prescription of hypoglycemic, vasodilator, or hormone replacement medications being nonpermissible. Those with ≤2 MetS were excluded at screening. Statin therapies and antihypertensive medications were allowed after habituation (i.e., antihypertensive medication, ≥6-mo; statins, ≥3-mo).

A hundred thirty-eight eligible participants were randomly assigned to 1 of 3 treatment groups, as previously described [18]. For this process, we used version 1.0 of the AR2007 covariate adaptive randomization allocation software devised by Kang and Park [39], which accounted for 4 balancing strata considered a priori likely to affect insulin resistance (the study’s main primary end point): sex, number of MetS criteria, age and statin/blood pressure medication use. The 6-mo intervention followed immediately after a 3-wk period of dietary restrictions (low in anthocyanin and flavonoids). The study treatments were as follows: 2 dietarily achievable blueberry intakes (equivalent to 1 and ½ United States cups of fresh blueberries/d, derived from homogenized, milled, and freeze-dried blueberries) and a matched isocaloric placebo powder.

**FIGURE 1:**
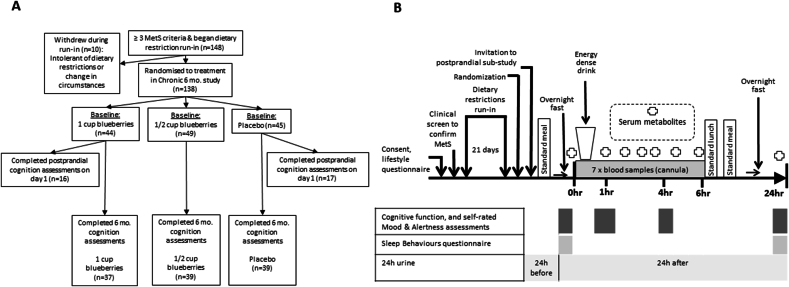
Flow chart of study participants and the content and timings for mood, alertness, and cognitive function assessments. (A) shows the recruitment and retention in the 6-mo study and those completing the cognitive assessments in the postprandial substudy at baseline; (B) shows the content and timing of the mood, alertness, and cognitive function assessments. MetS, metabolic syndrome.

The primary outcome for the 6-mo study, i.e., change (Δ 0–6 mo) in insulin resistance (assessed using the HOMA-IR), has been reported previously [18]. Presented here are the composite cognitive function data and the self-rated mood and alertness assessments, which were collected as secondary outcomes. These data were collected using a standardized battery of computerized cognitive function tests (the Cognitive Drug Research test battery; Bracket Global Limited), which was adapted to include Bond-Lader visual analog scales [40] for tests of mood and alertness. The Leeds Sleep Evaluation Questionnaire [41], an assessment of sleep quality, was also incorporated into the test battery. Because the apolipoprotein E (*APOE)* genotype has been associated with differential brain structure and performance throughout the life course [42], we assessed the status of all participants included in these analyses. Blueberry metabolite concentrations in urine and blood were assessed by liquid chromatography-mass spectrometry/mass spectrometry, as described previously [17,18], and the association between metabolites and cognitive function was determined in an exploratory, secondary analysis. The number and flow of participants completing this cognitive assessment (by treatment group) are shown in Figure 1A.

A postprandial substudy was also conducted at baseline, with 45 participants “opting-in” from the 1 cup blueberry and placebo groups (*n* = 23, 1 cup blueberry; *n* = 22, placebo: the ½ cup group were not invited to participate). This placebo-controlled, single-dose assessment tested the effect of incorporating the equivalent of ∼1 cup blueberries (i.e., 150 g, presented in powder form) alongside a high-fat, high-sugar meal, on postprandial cognitive and cardiometabolic responses – which were assessed at prescheduled times (see Figure 1). The protocol for the postprandial substudy, which provided an energy-dense meal challenge (969 kcals, 64.5 g fat, 84.5 g carbohydrate, 17.9 g protein), has been previously described [17]. As shown in Figure 1B, cognitive function, mood, alertness, and sleep quality were assessed at baseline (preceding the energy-dense drink intake) and then at +1, +4, and +24 h after the test drink consumption was initiated. This substudy required participants to attend the clinical facility for an extended period on 2 consecutive days (∼10 h on day 1, ∼3 h on day 2), have blood pressure continually monitored, and provide all the urine they passed over the 24-h collection period.

The study was approved by the National Research Ethics Committee (East of England), conducted at the National Health Service (NHS) Clinical Trials facility, University of East Anglia, United Kingdom, and completed between January 2014 and November 2016. The study was registered at www.clinicaltrials.gov (NCT02035592), followed the principles of the Declaration of Helsinki of 1975 (as revised in 1983), and participants gave written consent before enrollment.

### Dietary restrictions, intervention products, and compliance

For a 3-wk analyzed period, and throughout the study, participants were asked to restrict the intake of anthocyanins (completely abstaining from blueberry and restricting other anthocyanin-rich foods to 1 portion/wk) and foods known to modify vascular function (including tea, coffee, chocolate, red wine, and oily fish). A more extensive overview of the dietary restrictions in this study has been published previously [18]. To monitor dietary adherence, a 131-item validated food frequency questionnaire [43] was collected at baseline and then repeated at months 3 and 6. Additionally, food and drinks rich in anthocyanin, nitrate/nitrite, caffeine, or alcohol were avoided for 24 h before each assessment visit. In preparation for each assessment visit, bottled water (low in nitrite/nitrate) was consumed for 24 h, and an anthocyanin-free and low-flavonoid evening meal (standardized between volunteers) was provided.

Participants were randomly assigned to consume intervention products daily for 6-mo, with compliance calculated from returned wrappers and unused sachet counts. All 3 study treatments were provided within opaque, 26 g single-serve sachets, which were of similar appearance, taste, and texture. Treatment identity was masked (participants and study scientists), and an assessment of treatment blinding was assessed by questionnaire at 6 mo. The intervention treatments were isocaloric and matched for carbohydrate content (glucose 31%, fructose 30%, and sucrose 0%) and provided in a powdered, freeze-dried form. The products were consumed as foods, incorporated within existing diets and eating regimens, and consisted of a 26 g freeze-dried blueberry sachet (equivalent to 1 cup fresh blueberry; 364 mg anthocyanin and 879 mg phenolics), a hybrid treatment sachet that combined 13 g freeze-dried blueberries and 13 g placebo material (equivalent to ½ cup fresh blueberry; 182 mg anthocyanin and 439 mg phenolics), and a 26 g placebo sachet (0 mg anthocyanin and phenolics; produced by the National Food Lab).

### Assessment of mood and alertness, sleep evaluation, and cognitive function

A single computerized test battery was formulated (by Bracket Global Limited) to present tests in the following order: *1*) mood and alertness, *2*) sleep, and *3*) cognitive function. This sequence was chosen to minimize carry-over effects, as it was considered a priori that cognitive function testing would likely influence self-rated mood and alertness tests. To reduce the likelihood of a familiarization/training effect and reduce test-associated anxieties, each participant completed 2 training sessions with the Cognitive Drug Research equipment (at a separate visit) prior to their baseline assessment. In the postprandial substudy, the test battery was repeated at +1, +4, and +24 h after the high-fat, high-sugar challenge meal.

Bond-Lader Visual Analog Scale, which required participants to move a positional slider along a nondemarcated line to indicate their degree of self-rated “*alertness*,” “*calmness,*” and “*contentment*,” were collected at the beginning of every test battery assessment (chronic and postprandial). Conversely, the Leeds Sleep Evaluation Questionnaire, which assessed “*getting to sleep*,” “*quality of sleep*,” “*awakening from sleep,*” and “*behavior following wakefulness,*” were only presented within test batteries that followed a sleep cycle (i.e., fasted assessments on chronic days and the +24 h postprandial substudy assessment). The cognitive function element of the test battery included 10 discreet tests, which were consecutively executed over a ∼25-min period in a standardized manner under quiet conditions. The tests were as follows: *Simple Reaction Time*, *Digit Vigilance*, *Choice Reaction Time*, *Numeric Working Memory*, *Spatial Working Memory*, *Immediate Working Recall*, *Delayed Word Recall*, *Word Recognition*, *Picture Recognition*, and an *Executive Function Task*. Following manufacturer-standardized methods, the outputs from the cognitive function tests were assimilated into composite domains and outputs, i.e., *Attention* (i.e., power of attention, cognitive reaction time, continuity of attention, and reaction time variability), *Working Memory* (i.e., quality of working memory), *Episodic Memory* (i.e., quality of episodic memory), *Working and Episodic Memory* (i.e., quality of memory), *Speed of Retrieval from Memory* (i.e., speed of memory), *Executive Function*, and *Picture Recognition* (i.e., original stimuli accuracy, new stimuli accuracy).

### Laboratory analyses

We collected a 10 mL EDTA blood sample, from which we isolated the buffy layer that was subsequently stored at –80°C. Genotype SNP services (rs429358 and rs7412) from DNA extracted from the buffy coat layer were provided by LGC (LGC Biosearch Technologies) using a commercial gas-liquid chromatography SNPline plate-based genotyping workflow. The resultant data was used to provide *APOE* genotype (*E2/E2*, *E2/E3*, *E2/E4*, *E3/E3*, *E3/E4*, and *E4/E4*). For the purposes of the analysis, *APOE4* carrier status is defined as any of the following: *E2/E4*, *E3/E4*, and *E4/E4.* We determined the concentration of anthocyanin-derived phenolic acids in serum and urine samples (i.e., 24 h urine samples and sequential urine aliquots passed within the clinical facility during the postprandial substudy) using our established HPLC and mass spectrometer methodologies [17,18,44]. The concentrations of 72 metabolites were quantified.

### Statistical analysis

This was a secondary analysis of our main study [18], which was statistically powered for changes in insulin resistance. Although an absence of cognitive data in MetS after blueberry intake precluded a formal power calculation, we enrolled *n* ≥ 37/intervention group, which was marginally more than a recent study in 61 older adults [45] and almost double those recruited in similar parallel studies [25,29]. Using a retrospective power calculation (G∗Power software; https://www.psychologie.hhu.de/arbeitsgruppen/allgemeine-psychologie-und-arbeitspsychologie/gpower [46]), we established that a time-by-treatment interaction for *executive function* had 95% power with *n* = 39, *n* = 39 and *n* = 37 participants in the placebo, ½ cup and 1 cup blueberry groups, respectively. The effect of chronic intervention intake (i.e., daily intake, from 0–6 mo) on cognitive function, mood and alertness, and sleep quality was analyzed using a linear mixed-effect model where *time point* and the *treatment*-by-*time point* interaction were included as fixed factors, *participants* included as random effects, and *age* (y), *sex* (M/F), and *APOE4 carrier status* (Y/N) included as covariates. In the postprandial substudy, we used a linear mixed-effect model with time point (0 h, 1 h, 4 h, and 24 h), and the *treatment*-by-*time point* interaction included as fixed factors, *participants* included as random effects and *age* (y), *sex* (M/F), and *APOE4 carrier status* (Y/N) included as covariates. Nonnormally distributed data (assessed using the Skegness-Kurtosis test) were analyzed using a generalized linear model with a link log function. The postprandial model was similarly adjusted for *age*, *sex, APOE4 carrier status,* and *baseline value.* For the postprandial substudy, a retrospective power calculation was established, demonstrating that the time-by-treatment interaction for *calmness* had 69% power with *n* = 17 and *n* = 16 participants in the placebo and 1 cup blueberry groups, respectively (GLIMMPSE software https://glimmpse.samplesizeshop.org/#). To account for multiple testing, we separately calculated false discovery rate-adjusted *P* values using the Benjamin–Hochberg procedure (reported as Q values) for each of the types of data that we assessed, i.e., cognitive function, mood, and sleep. These false discovery rate data are displayed alongside the unadjusted *P* values..

An unadjusted, exploratory correlation analysis was performed to determine whether metabolite concentrations that increased following blueberry intervention (as we have previously published [18]) were associated with cognitive function, mood and alertness, and sleep quality. These analyses included both blueberry groups in the chronic study (1 cup and ½ cup) so that potential candidate metabolite targets linked to cognitive function could be identified. In these analyses, Spearman’s Rank Order (nonparametric) correlation was used to determine a correlation coefficient (*R* =). In the postprandial study, urinary metabolite concentrations were aligned with baseline levels so that negative and positive values represented decreases and increases in metabolites, compared with preintervention levels, respectively. A more detailed overview of the postprandial metabolite data assimilation has been described previously [17].

Data were analyzed using Stata version 16 (StataCorp LLC). *P* values of ≤0.05 were considered statistically significant.

## Results

Hundred fifteen participants completed the 6 mo study (*n* = 37, *n* = 39, *n* = 39; 1, ½ cup blueberries and placebo), with 33 of these participants (*n* = 17 placebo; *n* = 16 in the 1-cup blueberry group) also completing the baseline 24 h postprandial substudy (reported previously [17]) with complete cognitive function, mood, and alertness data, respectively. The MetS study population was predominantly obese (mean ± SD: BMI; 31.2 ± 3.0), mid to older aged (mean ± SD: age; 62.8 ± 7.1 y), and predominantly male (67.8%) with 24.3% being *APOE4* carriers (see Table 1). Over the 6-mo intervention period, there were no significant differences (*P* ≤ 0.05) in any cognitive function domain, although consumption of 1 cup of blueberries resulted in a trend toward a 4.2% improvement in percentage accuracy for picture recognition (*P* = 0.10; q = 0.59) (see Table 2). Similarly, the change in self-rated scores for alertness and mood did not differ by intervention group following chronic blueberry intake (*P* > 0.05), with only *alertness* (*P* = 0.08; q = 0.24) approaching significance following intake of 1 cup a day (see Table 2).

**TABLE 1:** Baseline characteristics of the 115 adults completing the 6-mo multidose blueberry intervention trial and the 33 adults completing the postprandial substudy^1^

	Chronic 6 mo study (*n* = 115)			Postprandial substudy (*n* = 33)	
	Placebo (*n* = 39)	½ Cup blueberries (*n* = 39)	1 Cup blueberries (*n* = 37)	Placebo (*n* = 17)	1 Cup blueberries (*n* = 16)
Age, y	62.9 ± 8.1	62.6 ± 7.2	63.0 ± 5.9	63.0 ± 9.1	64.5 ± 6.1
Sex (M), n (%)	28 (66.7)	28 (71.8)	24 (64.9)	10 (62.5)	11 (64.7)
BMI, kg/m^2^	31.1 ± 3.0	31.2 ± 2.6	31.3 ± 3.4	32.1 ± 2.9	31.3 ± 3.0
*APOE* 4 carrier status, n (%)	9 (29.4)	9 (25.0)	10 (30.3)	4 (25.0)	5 (29.4)
Power of attention, ms	1288 ± 131	1296 ± 143	1280 ± 211	1340 ± 154	1341 ± 288
Continuity of attention, ms	92.0 ± 2.9	91.1 ± 3.2	91.4 ± 3.5	92.0 ± 2.8	90.6 ± 4.3
Cognitive reaction time, ms	209 ± 51.9	206 ± 52.0	198 ± 36.7	220 ± 53.1	209 ± 40.1
Reaction time variability, %	46.3 ± 9.4	47.9 ± 10.8	45.8 ± 8.5	44.5 ± 7.2	47.5 ± 9.8
Quality of working memory, SI	1.8 ± 0.3	1.7 ± 0.3	1.9 ± 0.1	1.8 ± 0.4	1.8 ± 0.1
Quality of episodic memory, #	176 ± 48.1	165 ± 49.2	176 ± 52.5	184 ± 45.6	167 ± 44.9
Quality of memory, #	355 ± 60.0	338 ± 61.9	361 ± 57.4	361 ± 72.0	348 ± 49.6
Speed of memory, ms	3908 ± 734	4190 ± 1050	3992 ± 603	3901 ± 854	4134 ± 658
Executive function score, #	0.1 ± 0.0	0.1 ± 0.0	0.1 ± 0.0	0.1 ± 0.0	0.1 ± 0.0
Picture recognition original stimuli accuracy, %	89.3 ± 10.7	92.3 ± 9.7	92.8 ± 8.0	91.9 ± 8.5	88.8 ± 9.3
Picture recognition new stimuli accuracy, %	88.1 ± 8.7	85.0 ± 12.7	87.8 ± 12.2	89.7 ± 11.5	87.9 ± 9.9
Bond-Lader self-rated alertness, mm	69.7 ± 15.3	67.8 ± 13.2	71.5 ± 15.8	68.5 ± 12.0	67.5 ± 13.8
Bond-Lader self-rated contentment, mm	79.7 ± 15.2	79.5 ± 14.4	82.1 ± 13.8	78.5 ± 10.6	77.2 ± 17.9
Bond-Lader self-rated calmness, mm	67.8 ± 17.1	67.1 ± 17.6	70.8 ± 16.2	68.6 ± 12.9	65.8 ± 16.8

Abbreviations: APOE, apolipoprotein E; BMI, body mass index; CDR, Cognitive Drug Research; M, male; SD, standard deviation; SI, sensitivity index value, generated by the CDR test battery system.

1 Mean ± SD (all such values).

**TABLE 2:** Change in cognitive, mood, and sleep outcomes from baseline to 6 mo by intervention group^1^, ^2^, ^3^

	Δ 0–6 mo in placebo (*n* = 39)	Δ 0–6 mo ½ cup (*n* = 39)	Δ 0–6 mo 1 cup (*n* = 37)	*P* value	q value
Power of attention, msec	14.7 (–9.72, 39.1)	27.6 (3.6, 51.6)	37.0 (11.9, 62.2)	0.43	0.62
Continuity of attention, msec	0.12 (–0.87, 1.1)	–0.33 (–1.29, 0.63)	0.76 (–0.26, 1.8)	0.31	0.59
Cognitive reaction time, msec	–11.35 (–27.55, 4.8)	6.3 (–9.65, 22.3)	1.4 (–15.07, 17.8)	0.29	0.59
Reaction time variability, %	0.17 (–2.55, 2.9)	0.35 (–2.28, 3.0)	3.4 (0.69, 6.2)	0.17	0.59
Quality of working memory, SI	0.01 (–0.07, 0.09)	0.00 (–0.07, 0.07)	0.02 (–0.06, 0.10)	0.93	0.93
Quality of episodic memory	26.1 (10.4, 41.7)	22.9 (8.2, 37.6)	17.1 (1.7, 32.5)	0.72	0.88
Quality of memory	30.9 (10.4, 51.5)	8.9 (–10.20, 28.0)	14.4 (–5.78, 34.6)	0.32	0.59
Speed of memory, msec	–67.99 (–207.02, 71.0)	55.2 (–79.92, 190)	9.5 (–125.77, 145)	0.45	0.62
Executive function score	0.002 (–0.003, 0.007)	–0.003 (–0.007, 0.001)	0.001 (–0.004, 0.005)	0.30	0.59
Picture recognition original stimuli accuracy, %	–0.75 (–3.96, 2.5)	1.3 (–1.79, 4.4)	4.2 (0.91, 7.4)	0.10	0.59
Picture recognition new stimuli accuracy, %	1.2 (–2.26, 4.6)	1.3 (–1.98, 4.6)	0.23 (–3.21, 3.7)	0.89	0.93
Bond-Lader self-rated alertness, mm	–5.53 (–10.25, –0.81)	–6.03 (–10.83, –1.24)	0.94 (–3.86, 5.7)	0.08	0.24
Bond-Lader self-rated contentment, mm	–2.18 (–6.15, 1.8)	–3.61 (–7.57, 0.36)	0.01 (–3.94, 4.0)	0.44	0.66
Bond-Lader self-rated calmness, mm	1.1 (–4.50, 6.8)	2.4 (–3.20, 8.1)	1.7 (–4.03, 7.4)	0.95	0.95
LSEQ-behavior following wakefulness, mm	–2.56 (–7.21, 2.1)	–1.31 (–5.90, 3.3)	–5.48 (–10.21, –0.76)	0.45	0.45
LSEQ getting to sleep, mm	0.73 (–0.38, 1.8)	–0.40 (–1.47, 0.67)	–0.49 (–1.62, 0.63)	0.23	0.45
LSEQ awakening from sleep, mm	0.18 (–0.47, 0.83)	0.67 (0.04, 1.3)	–0.09 (–0.76, 0.58)	0.26	0.45
LSEQ quality of sleep, mm	–0.90 (–1.98, 0.17)	0.20 (–0.85, 1.3)	–0.28 (–1.39, 0.82)	0.35	0.45

Abbreviations: APOE, apolipoprotein E; CI, confidence interval; CDR, Cognitive Drug Research; LSEQ, Leeds Sleep Evaluation Questionnaire; mm, millimeters of measurement on visual analog scales; SD, standard deviation; SI, sensitivity index value, generated by the CDR test battery system.

1 Values are mean (95% CI) adjusted for age, sex, and *APOE 4* carrier status. Outliers < or > 3.5 SD were excluded (i.e., *n* = 1 for *cognitive reaction time*, *continuity of attention*, *quality of working memory*, *quality of memory*, *picture recognition original stimuli accuracy*, *LSEQ-behavior following wakefulness*, and *Bond-Lader self-rated alertness*; *n* = 2 for *executive function score*, *power of attention*, *LSEQ awakening from sleep*, and *Bond-Lader self-rated contentment*; and *n* = 3 for *speed of memory*, *LSEQ getting to sleep*, and *LSEQ quality of sleep*.

2 Cup relates to the equivalent number of United States cups of fresh blueberries.

3 *P = P* values for the time x treatment interaction. q value = false discovery rate-adjusted *P* values for the time x treatment interaction.

A lack of benefit on cognitive function, alertness, and sleep quality was also observed in the postprandial substudy, with no effect of blueberries evident across the 24 h period (*P* > 0.05) (Table 3). The exception to this was the observation that participants self-rated their degree of *calmness* significantly higher after 1 cup of blueberry intake compared with placebo (*P* = 0.01; q = 0.04) – with differences in the extent of *calmness* being the greatest at the 24 h time point (Table 3). When exploring the association between changes in metabolite levels and potentially favorable cognitive function, mood and alertness, and sleep quality indices, there were *n* = 10 associations (*P* ≤ 0.05) following chronic (0–6 mo) and *n* = 11 associations (*P* ≤ 0.05) following postprandial intake of blueberries and markers of cognitive function (Table 4). As shown in Table 4, we observed moderate chronic associations (*R* = ±0.24 to ±0.37; *P* < 0.001 to *P* = 0.05) and postprandial associations (*R* = ±0.45 to ±0.61; *P* = 0.01 to *P* = 0.05) across urinary and serum metabolites and assessments, including memory (i.e., *speed*, *working*, *episodic*, *recognition*, and *continuity*), attention (i.e., *continuity*, *power*, *alertness*, and *wakefulness*), as well as *executive function* and *calmness*. The metabolite groups most notably associated were microbial metabolites of anthocyanins and chlorogenic acid (i.e., *hydroxycinnamic acids*, *benzoic acids*, *phenylalanine derivatives,* and *hippuric acids*) and catechin.

**TABLE 3:** Baseline adjusted cognitive, mood, and sleep outcomes, assessed between 0 and 24 h after the intake of an energy-dense test meal challenge, with/without 1 cup of blueberries (postprandial study)^1^, ^2^, ^3^

	Placebo (*n* = 17)				1 cup (*n* = 16)					
	0	1 h	4 h	24 h	0	1 h	4 h	24 h	*P* value	q value
Power of attention, msec	1330 (1238, 1423)	1378 (1283, 1474)	1346 (1253, 1440)	1315 (1223, 1406)	1289 (1199, 1378)	1363 (1269, 1457)	1330 (1238, 1422)	1419 (1321, 1516)	<0.001	<0.001
Continuity of attention, msec	91.9 (90.4, 93.4)	91.9 (90.4, 93.4)	92.3 (90.8, 93.8)	93.3 (91.9, 94.8)	90.6 (89.1, 92.0)	91.1 (89.6, 92.5)	90.5 (89.1, 92.0)	90.6 (89.1, 92.1)	0.39	0.61
Cognitive reaction time, msec	220 (201, 240)	219 (200, 238)	200 (182, 218)	190 (172, 207)	204 (186, 223)	192 (175, 210)	193 (176, 211)	194 (176, 212)	0.14	0.51
Reaction time variability, %	44.0 (40.4, 47.7)	47.1 (43.3, 50.9)	48.7 (44.9, 52.4)	45.7 (42.0, 49.3)	47.1 (43.4, 50.8)	50.8 (46.9, 54.6)	47.5 (43.8, 51.2)	51.0 (47.1, 54.9)	0.09	0.51
Quality of working memory, SI	1.8 (1.6, 1.9)	1.8 (1.7, 2.0)	1.8 (1.6, 1.9)	1.8 (1.7, 1.9)	1.8 (1.7, 1.9)	1.7 (1.6, 1.8)	1.6 (1.4, 1.7)	1.7 (1.6, 1.9)	0.30	0.61
Quality of episodic memory	181 (163, 199)	135 (117, 154)	139 (121, 157)	159 (141, 177)	169 (151, 187)	138 (120, 156)	143 (125, 161)	153 (135, 171)	0.56	0.76
Quality of memory	356 (329, 383)	315 (290, 341)	312 (287, 337)	337 (311, 362)	345 (319, 371)	307 (283, 332)	297 (273, 321)	324 (298, 350)	0.98	0.98
Speed of memory, msec	3905 (3592, 4219)	3801 (3494, 4108)	3820 (3512, 4128)	3716 (3417, 4014)	4036 (3718, 4353)	4071 (3751, 4390)	4170 (3845, 4494)	4119 (3794, 4443)	0.38	0.61
Executive function score	0.12 (0.11, 0.13)	0.11 (0.10, 0.12)	0.12 (0.11, 0.13)	0.13 (0.12, 0.13)	0.11 (0.10, 0.12)	0.11 (0.10, 0.11)	0.11 (0.10, 0.12)	0.11 (0.11, 0.12)	0.92	0.98
Picture recognition original stimuli accuracy, %	91.1 (86.4, 95.8)	89.3 (84.7, 94.0)	86.4 (81.9, 90.9)	90.5 (85.9, 95.1)	88.6 (84.1, 93.2)	86.4 (81.9, 90.8)	89.6 (85.0, 94.1)	88.2 (83.5, 92.8)	0.20	0.55
Picture recognition new stimuli accuracy, %	89.2 (85.0, 93.3)	86.0 (81.9, 90.1)	86.1 (82.1, 90.1)	89.7 (85.6, 93.7)	88.4 (84.3, 92.4)	87.2 (83.2, 91.2)	87.5 (83.5, 91.5)	90.8 (86.6, 94.9)	0.90	0.98
Bond-Lader self-rated alertness, mm	68.7 (62.2, 75.1)	65.3 (59.1, 71.6)	66.6 (60.3, 72.8)	69.1 (62.7, 75.5)	66.1 (59.9, 72.3)	66.7 (60.5, 73.0)	63.9 (57.8, 70.0)	70.7 (64.1, 77.3)	0.48	0.48
Bond-Lader self-rated contentment, mm	78.7 (71.6, 85.7)	76.8 (69.9, 83.8)	77.9 (71.0, 84.9)	75.6 (68.8, 82.4)	75.8 (69.0, 82.6)	76.5 (69.7, 83.3)	75.8 (69.0, 82.6)	78.7 (71.6, 85.7)	0.29	0.43
Bond-Lader self-rated calmness, mm	69.1 (62.4, 75.7)	72.7 (66.0, 79.3)	72.9 (66.4, 79.4)	64.7 (58.2, 71.3)	65.6 (59.0, 72.1)	70.7 (64.2, 77.3)	68.9 (62.3, 75.4)	73.2 (66.5, 79.8)	0.01	0.04
LSEQ awakening from sleep, mm	49.8 (49.6, 50.0)	-	-	50.1 (49.8, 50.3)	50.1 (49.8, 50.3)	-	-	49.9 (49.7, 50.2)	0.08	0.17
LSEQ-behavior following wakefulness, mm	57.3 (50.3, 64.3)	-	-	57.2 (50.3, 64.0)	61.9 (54.8, 69.0)	-	-	57.6 (50.7, 64.6)	0.41	0.41
LSEQ getting to sleep, mm	48.9 (48.2, 49.7)	-	-	49.4 (48.7, 50.1)	49.7 (49.0, 50.4)	-	-	49.4 (48.7, 50.1)	0.24	0.32
LSEQ quality of sleep, mm	49.7 (49.5, 49.9)	-	-	49.9 (49.7, 50.2)	50.2 (49.9, 50.4)	-	-	49.9 (49.7, 50.2)	0.03	0.12

Abbreviations: APOE, apolipoprotein E; CI, confidence interval; CDR, Cognitive Drug Research; LSEQ, Leeds Sleep Evaluation Questionnaire; mm, millimeters of measurement on visual analog scales; SI, sensitivity index value, generated by the CDR test battery system.

1 Values are mean (95% CI) adjusted for age, sex, and *APOE4* carrier status.

2 *P* values for the time point x treatment interaction were calculated using a constrained linear mixed-effect model (adjusting for baseline values).

3 q value = false discovery rate-adjusted *P* values for the time x treatment interaction.

**TABLE 4:** Exploratory correlations between metabolite and cognitive outcomes in blueberry-treated participants (*n* = 16 postprandial; *n* = 76 chronic study)^1^, ^2^, ^3^

Cognitive outcome	Metabolite	Timeframe	Biospecimen	*r*	95% CI	*P* value
Attention						
Power of attention, msec	3-hydroxybenzoic acid	Chronic	Urine	–0.36	(–0.56, –0.13)	<0.001
	trans-3-hydroxycinnamic acid	Chronic	Urine	–0.25	(–0.47, –0.00)	0.05
	hydroxymethoxybenzoic acid-sulfate^1^	Postprandial	Serum	–0.49	(–0.77, –0.04)	0.04
Continuity of attention, msec	3,4-dihydroxyphenylacetic acid	Chronic	Urine	0.25	(0.00, 0.47)	0.05
	benzoic acid-4-sulfate	Postprandial	Urine	0.51	(0.06, 0.79)	0.03
Memory						
Quality of working memory, SI	3-methoxybenzoic acid-4-sulfate and 4-methoxybenzoic acid-3-sulfate^2^	Chronic	Serum	0.37	(0.16, 0.55)	<0.001
Quality of episodic memory	3-methoxyphenylacetic acid-4-sulfate	Postprandial	Serum	0.49	(0.03, 0.78)	0.04
	3-hydroxyhippuric acid	Postprandial	Urine	0.48	(0.00, 0.78)	0.05
Speed of memory, msec	3,4-dihydroxyphenylacetic acid	Chronic	Urine	–0.30	(–0.51, –0.05)	0.02
	3-O-caffeoylquinic acid (chlorogenic acid)	Postprandial	Urine	–0.49	(–0.77, –0.05)	0.03
	hippuric acid	Postprandial	Serum	–0.46	(–0.76, –0.01)	0.05
Executive function						
Executive function score	4-hydroxybenzoic acid	Postprandial	Urine	0.61	(0.22, 0.84)	0.01
	benzoylglutamic acid	Postprandial	Urine	0.60	(0.21, 0.83)	0.01
Picture recognition						
Picture recognition original stimuli accuracy, %	hippuric acid	Chronic	Urine	0.27	(0.03, 0.49)	0.03
	3-hydroxyhippuric acid	Chronic	Urine	0.26	(0.01, 0.47)	0.04
Self-rated mood and alertness						
Alertness, mm	4-hydroxy-3,5-dimethoxyphenylacetic acid	Chronic	Serum	0.24	(0.00, 0.46)	0.05
Calmness, mm	3-hydroxy-4-methoxycinnamic acid	Chronic	Urine	0.28	(0.03, 0.50)	0.03
Sleep behaviors						
Wakefulness, mm	4-hydroxy-3,5-dimethoxyphenylacetic acid	Chronic	Serum	0.26	(0.03, 0.47)	0.03
	3-(4-hydroxy-3-methoxyphenyl) propionic acid	Postprandial	Serum	0.45	(0.00, 0.75)	0.05
Quality of sleep, mm	3-methoxyphenylacetic acid 4-sulfate	Postprandial	Serum	0.61	(0.21, 0.83)	0.01
	3-hydroxy-4-methoxyphenylacetic acid	Postprandial	Serum	0.45	(–0.01, 0.75)	0.05

Abbreviations: CDR, Cognitive Drug Research; CI, confidence interval; mm, millimeters of measurement on visual analog scales; MS/MS, mass spectrometry/mass spectrometry; SI, sensitivity index value, generated by the CDR test battery system.

1 r = correlation coefficient from Spearman’s Rank Order Correlation (nonparametric correlation); adjusted for treatment group.

2 Putative identification of methylgallic acid-sulfate: no analytical reference standard was available for the compound having matched MS/MS fragmentation pattern to methylgallic acid (Dihydroxy-methoxybenzoic acid) and having a neutral loss of 80 m/z (indicative of conjugation with sulfate).

3 Isomers could not be resolved and were quantified as a single peak.

## Discussion

To our knowledge, in the first long-term assessment of cognitive function, mood, and alertness in adults with MetS, we report that chronic consumption of blueberries, at either 1 or ½-cup intakes/d for 6 mo, had no significant benefits on the outcomes we assessed. Similarly, in adults with MetS who consumed a high-fat, high-sugar energy-dense test drink, the inclusion of 1 cup of blueberries did not meaningfully alleviate the expected postprandial declines in cognitive function, mood and alertness, with the exception of participant-rated levels of *calmness*, which peaked at the 24 h period after inclusion of blueberries. Considering that our earlier analyses, as part of this randomized controlled trial, had confirmed that 1 cup of blueberries significantly improved cardiometabolic health both chronically [18] and postprandially [17], these current data do not support a coalescence between beneficial shifts in cardiometabolic health, cognition domains and self-rated mood and alertness in MetS.

Our results contrast with previous studies in those with cognitive impairments or subjective cognitive decline, which have shown that 3–6 mo of blueberry intake improved cognitive domains, including processing speed [24], executive function [25], memory impairment, and verbal span [26], and everyday activities which relied on manifestations of cognition and memory functions [25,27]. Blueberry intake also increased blood oxygenation level-dependent activation in MCI (in the left precentral gyrus, middle frontal gyrus, and inferior parietal lobe) during working memory load tasks, indicating enhanced neuronal activation [28]. In addition to existing cognitive decline potentially providing a window of intervention opportunity for benefit, the reason for these disparities may, in part, be explained by heterogeneity in study design and participant characteristics that may conceivably influence cognitive performance, including the cognitive assessment protocols used, participant age, and differences in cognitive functionality of study participants (and the likely differential underlying pathophysiologies).

Perhaps uniquely, 1 of these recent studies [24] intervened with freeze-dried wild blueberries for an identical intervention period (6 mo) in cognitively declining older seniors (established via Montreal Cognitive Assessment scoring), which provides a novel counterpoint to explore why differential cognitive responses were found between those with, and without, cognitive dysfunction. Comparatively, our study population was younger (mean age ∼63 y), had a normal cognitive function, and undertook a shorter and less-arduous test battery (i.e., 25 min) than in the study by Cheatham et al. [24]. In the latter study, improvements in speed of processing (latency) assessments (rapid visual processing test) were supported by tandem shifts in electro-physically monitored brain regions in older seniors (75–80 y), but interestingly, not those aged 65–69 y [24]. In this study, cognitive benefits of blueberries were only apparent in those experiencing the highest cognitive load, i.e., *1*) the oldest (75–80 y), *2*) with pre-existing cognitive dysfunction, and *3*) during the most cognitively challenging test (i.e., rapid visual processing), scheduled 3 h into a sustained cognitive test battery. Similarly, it has been shown that the combined cognitive benefits of omega-3 fatty acids, carotenoids, and vitamin E oils were only discernible (in those without cognitive dysfunction) during cognitive challenges of increased complexity and duration [47]. Together, these data support that cognitive benefits of nutritional intervention may only manifest above a threshold of cognitive demand/load, suggesting a role for yet-to-be-confirmed mechanisms that alleviate stress response pathophysiologies. On the basis of a combination of in vitro, animal, and human data, we propose that such mechanisms may include the attenuation of neuroinflammation [48,49], mitochondrial allostatic load [50], and buffering of mental stress-induced vascular reactivity as recently shown following acute, dark chocolate intake [51].

In populations without cognitive dysfunction, there has, to date, been equivocal data for the benefits of blueberries. Although verbal learning appears enhanced following short-chronic blueberry intakes in healthy populations (i.e., 3 mo [[29], [30], [31]]), these effects were not observed at the 6 mo repeat assessment in the study by Whyte et al. [31], highlighting that apparent shorter-term improvement may not accurately reflect the likelihood of sustained cognitive adaptations. Likewise, domains, including reaction times, episodic memory, working memory, spatial memory, and executive function have all been unaffected by blueberry intake in cognitively unaffected populations [[30], [31], [32], [33]]. In our study, only chronic (0–6 mo) *picture recognition accuracy of original stimuli* approached statistical significance (*P* = 0.10), with a somewhat graded improvement as the dose of blueberries increased from ½–1 cup/d. In this test, participants identified whether they had seen an image in a deck of images shown ∼20 min earlier, with visually similar (but not identical) images shown to distract their judgment. This test requires relatively short-term, synaptic/initial memory consolidation, which is well described as occurring in the hippocampal region – with recent evidence specifically identifying a key role of the amygdala [52]. Interestingly, numerous recent animal studies have shown adaptations in neuroinflammation and neuroplasticity of the hippocampal region following blueberry extract or blueberry (poly)phenol interventions [49,[53], [54], [55], [56]], which suggests this may be a priority area to focus on in future human studies.

To date, only studies in healthy adults (i.e., those without elevated cardiometabolic risk profiles, such as MetS) have assessed postprandial cognitive responses to blueberries, with cognitive benefits only attained during demanding and sustained tasks across this limited dataset [34,35]. As identified previously, our cognitive battery was relatively short (at 25 min) and may not have elicited the cognitive load required to observe effects. That accepted it was notable that our participants reported a significant increase in *calmness* across the postprandial period (0–24 h), with peak levels reported at the 24 h time point. Interestingly, recent animal studies have shown antidepressant-like qualities of blueberry extracts [49], and in human studies, blueberries have previously improved positive affect measures (although not negative affect measures), suggesting mood improvement [36]. However, these effects have not been unequivocally supported [37], and the effects of blueberries on mood and well-being require further confirmation.

We have previously shown that concentrations of anthocyanin-derived metabolites shifted as a consequence of both chronic [18] and postprandial [17] blueberry intake. In this assessment, designed to facilitate hypothesis generation, we performed an exploratory secondary analysis across the 2 blueberry groups (½ and 1 cup) to identify if the increases in blueberry-anthocyanin metabolites that we had previously observed were associated with shifts in cognitive function, mood or sleep quality – both chronically and postprandially. We observed that 12 metabolites following chronic feeding and 10 metabolites in the postprandial state were associated with potentially favorable changes in cognitive function, mood, or sleep quality (*P* ≤ 0.05). These associations were observed throughout metabolic pathways that have been previously established following berry intake, i.e., from *chlorogenic acid* (associated with the postprandial *speed of memory*) to *hydroxycinnamic acids* (associated with chronic *power, continuity of attention,* and *calmness*) to *benzoic acids* (associated with chronic *power, continuity of attention*, *working memory,* and postprandial *power of attention,* and *executive function)*; to *phenylalanine derivatives* (associated with chronic *speed of memory, alertness and wakefulness,* and postprandial *episodic memory*, *quality of sleep* and *wakefulness);* and finally to *hippuric acids* (associated with chronic *picture recognition of original stimulus,* and postprandial *speed of memory* and *episodic memory).* In previous human studies, *chlorogenic acid (3-O-caffeoylquinic acid)* improved the psychomotor, motor, and processing speeds of healthy midaged Japanese participants (50–65 y) over a 2 wk period [57] and reduced errors by those with cognitive dysfunction [58]. In cross-sectional analysis, it has also been shown that a higher intake of *hydroxycinnamic acids* is associated with improvements in cognitive status and sleep quality in older Italian adults [59,60]. Similarly, in a 90 d blueberry feeding trial, increases in *hydroxycinnamic acids* (*ferulic acid-glucuronide/3-methoxycinnamic acid-4-O-glucuronide*), *benzoic acids* (*syringic acid/4-hydroxy-3,5-dimethoxybenzoic acid*), and *hippuric acid* were associated with reductions in cognition errors (i.e., the California Verbal Learning Test, or the Task-Switching-Test) in healthy older adults [29]. As our data were exploratory and were not adjusted for multiple testing, we have been cautious in our interpretation - especially as no significant changes were elicited at the intervention group level. However, accepting those caveats, it was notable that the breadth of commonly produced metabolites of berry origin (i.e., specifically from blueberry, in our study) was relatively strongly associated with favorable cognition and mood outcomes (≤*R = 0.61*, *P < 0.001* levels). Moreover, in the absence of cognitive function effects at the group level, these data imply that our sample experienced differential metabolism outcomes, and those *responders* who produced more of these common metabolites had greater cognitive benefits, which reconfirms the need for greater personalization in nutritional interventions. From a public health perspective, it was also striking that these metabolite-cognition associations were not unique metabolites from single-food sources; thus, our data provide egalitarian metabolite targets for plant-based food interventions (especially fruit and berries) to cater to different tastes and accessibility. Future studies explicitly designed to investigate metabolite-cognition interactions are now required to confirm these data.

Our study had a number of strengths, including being a long-term intervention with multiple blueberry doses, which also included a postprandial substudy that provided a unique insight into the effect of blueberries on acute and chronic cognitive function in the same population with MetS. However, this *acute-on-chronic*, “opt-in” design also unblinded those in this substudy to 1 treatment (i.e., the ½ cup dose – which was not included in this substudy for capacity reasons), but participants remained blinded to either placebo or 1 cup group allocation. These participants also received 3 additional cognitive function assessments (at +1, +4, and +24 h after baseline assessment), compared with those not completing the postprandial study (across all treatment groups; placebo, ½, 1 cup groups). Although it is unknown whether these factors affected our outcomes, it was reassuring that there was relative parity in participant numbers within the postprandial cognitive assessment groups (*n* = 17, placebo; *n* = 16, 1 cup), and our 6 mo data do not suggest preferential improvements in cognition by any treatment group. A further limitation was that our study was powered to detect a change in the primary end point, chronic insulin resistance (as previously described [18]), and cognitive function, mood, and sleep were secondary outcomes that were not formally powered. Despite this, our chronic intervention groups (i.e., *n* = 37–39/group) were larger than other blueberry studies that were powered explicitly for cognitive function (i.e., [[25], [26], [27],[29], [30], [31]]), and we had similar numbers to comparable postprandial studies (e.g., [34,61]). These descriptive comparisons are supported by our retrospective analysis of statistical power [using G∗Power (chronic data) and GLIMMPSE (postprandial data) software], which identified relatively high power for key variables in our chronic (i.e., 95% power for executive function) and postprandial assessments (i.e., 69% power for calmness). Finally, despite an open call to recruit members of the general public, our population (across all 3 intervention groups), seemingly by chance, appeared to be performing above the expected normative range for the cognitive function testing (i.e., compared with normative data for % accuracy for picture recognition, using the same equipment and tests [62]), which may have limited the capacity for blueberry to improve cognition. Despite these limitations, this work has provided much-needed data for the cognitive effects of blueberry intake in those with MetS, establishing that our previously identified cardiometabolic benefits do not translate to improved cognitive function despite positive shifts in concentrations of anthocyanin-derived metabolites.

In summary, to our knowledge, we present the first evidence in those with MetS but without cognitive dysfunction that neither chronic nor postprandial blueberry intake was effective in improving cognition, mood, or sleep quality – with the exception of postprandial *calmness*. We suggest that our data reinforce the increasing evidence that the benefits of blueberries are more likely to be realized in those experiencing higher cognitive loads – such as those functioning under stressful situations or performing tasks with high cognitive demands, older seniors, or those with pre-existing cognitive dysfunction, and are an ineffective strategy for cognitive function improvement in those with MetS, but without cognitive dysfunction.

## Acknowledgments

We acknowledge the involvement of the Clinical Research Facility at the University of East Anglia (UEA), administrative assistance from Tim Greene (UEA), and recruitment and intervention assistance from Corbin Griffen (UEA).

## Data Availability

Anonymized data described in the manuscript will be made available upon request to the corresponding author, pending the submission and approval (by the corresponding author and team) of an appropriate research hypothesis and statistical analysis plan.
